# Implementing Robotic Gynecologic Surgery: Early Outcomes From a Tertiary Care Hospital in Portugal

**DOI:** 10.7759/cureus.89804

**Published:** 2025-08-11

**Authors:** Andreia Martins, Sara Moreira, Filipa Alpendre, Paula Ambrósio

**Affiliations:** 1 Gynecology, Unidade Local de Saúde de São José, Maternidade Dr. Alfredo da Costa, Lisboa, PRT; 2 Gynecologic Oncology, Unidade Local de Saúde de São José, Maternidade Dr. Alfredo da Costa, Lisboa, PRT

**Keywords:** gynecology, hysterectomy, minimally invasive surgery, robotic surgery, surgical outcomes

## Abstract

Introduction

Robotic surgery is increasingly used in minimally invasive gynecologic procedures. In Portugal, its integration into the National Health Service is still recent. This study presents the early clinical experience of a Portuguese tertiary center with robot-assisted gynecologic surgery.

Materials and methods

This was a retrospective, observational, and descriptive study based on the analysis of clinical charts from patients who underwent robot-assisted gynecologic surgery between October 2022 and December 2024. Demographic, intraoperative, and postoperative variables were evaluated, as well as histopathological results.

Results

A total of 109 patients underwent robot-assisted gynecologic surgery during the study period. The median patient age was 55 years, and the median BMI was 26.6 kg/m². The main surgical indication was adnexal tumor in 46 patients (42.2%), followed by endometrial tumor or its precursors in 33 patients (30.3%). The most commonly performed procedure was total hysterectomy (69.7%). The median operative time was 193 minutes, and estimated blood loss was less than 100 mL in most cases (66.1%). The conversion rate to laparotomy was 0.9%, and the median length of hospital stay was one day. Complications occurred in 10.1% of patients, most of which were Clavien-Dindo grade I or II. The readmission rate was 2.8%.

Conclusion

The implementation of robotic-assisted gynecologic surgery in our center was associated with favorable short-term outcomes, including low complication and readmission rates, minimal blood loss, and rapid recovery. While limited to a single-center experience, this study adds to the growing evidence on the safe and feasible introduction of robotic surgery in gynecologic practice.

## Introduction

Robotic surgery is an important innovation in minimally invasive surgery, allowing the surgeon to control the movement of the robot's arms with high precision through a console.

The initial milestone of robotics in medicine occurred in 1985 with the use of the PUMA 200 system, designed to perform neurosurgical biopsies in humans [1]. Subsequently, the robot was adapted to perform urological procedures, including prostate-related interventions, at the Robotics Center of the Imperial College (PROBOT system) [2]. In 1992, the ROBODOC® Surgical System was developed, an image-guided robotic system designed for total hip prosthesis procedures [3]. The ZEUS robotic system was introduced in 1998 by Computer Motion, marking the beginning of the use of telepresence in surgery. This platform included three independent arms. The ZEUS robotic platform was first used in 1998 at the Cleveland Clinic for a fallopian tube anastomosis surgery, standing out as the first global robotic application in gynecology [4].

In 1999, Intuitive Surgical Inc. launched the first Da Vinci® Surgical System. The goal was to develop more precise and minimally invasive techniques, providing enhanced anatomical visualization and improved surgical dexterity. In 2000, the system was approved by the Food and Drug Administration (FDA) for use in general laparoscopic surgery [5]. In 2005, the system was specifically approved by the FDA for use in gynecologic procedures, following successful myomectomies and hysterectomies at the University of Michigan [6].

In Portugal, robotic surgery was introduced in 2010 at a private hospital. Since then, its implementation has progressively expanded, driven by increasing demand and positive results. It is now available in numerous institutions across both the public and private sectors [7].

The Gynecology Department of Unidade Local de Saúde de São José (ULS São José), Maternidade Dr. Alfredo da Costa, initiated its robotic surgery program on October 18, 2022. The implementation of robotic surgery at our department followed a structured, multi-phase training program. This included theoretical education through the Intuitive Surgical Inc. platform, followed by an online knowledge assessment and three theoretical-practical sessions. Additionally, the team participated in live surgery observations at a specialized center and completed at least 40 hours of hands-on simulation training. The process concludes with final certification by Intuitive Surgical Inc., attesting to the surgical competencies acquired during the training program. The team currently includes two specialists in gynecologic oncology and two in urogynecology, all certified by Intuitive Surgical Inc.

The aim of this study is to report the experience of the Gynecology Department of ULS São José since the initiation of robotic surgery, describing the patients' demographic and clinical characteristics, as well as the intraoperative and postoperative outcomes achieved during the period.

This article was previously presented as an e-poster at the XVI Portuguese Congress of Gynecology, held from June 5 to 7, 2025.

## Materials and methods

Study design

A retrospective observational study was conducted on patients who underwent gynecologic surgery using a robotic approach at a tertiary center in Portugal, between October 18, 2022, and December 31, 2024.

Variables

Data were obtained from each patient's electronic medical record. Demographic variables analyzed included age, body mass index (BMI), American Society of Anesthesiologists (ASA) classification, comorbidities, previous surgeries, and surgical indication. Intraoperative variables included operative time, estimated blood loss, conversion to laparotomy, and intraoperative complications. Postoperative variables included length of hospital stay, readmission, complications occurring within the first 30 days (classified according to the Clavien-Dindo system), and need for surgical reintervention.

Technical approach

The procedures were performed using the Da Vinci X® HD and Xi® HD robotic surgical systems (Intuitive Surgical, Inc.). Surgeries were performed by members of the same surgical team, which included two specialists in gynecologic oncology and two in urogynecology. All surgeons completed the same structured, multi-phase training program prior to performing robotic procedures.

Statistical analysis

Data were entered using Microsoft Excel (Microsoft, Redmond, Washington), and statistical analysis was conducted using SPSS Statistics version 23 (IBM Inc., Armonk, New York). Descriptive statistical methods were used to summarize qualitative and quantitative variables. Qualitative variables were reported as absolute and relative frequencies (n, %), while quantitative variables were expressed as medians, minimum and maximum values (min-max), and interquartile range (IQR). The results are also presented in tables.

## Results

During the study period, 109 patients underwent surgery (Table 1). The median age was 55 years (range: 23-85), and the median BMI was 26.6 kg/m² (range: 16-49). Regarding comorbidities, 34 patients (31.2%) had hypertension, 10 (9.2%) had diabetes mellitus, and 71 (65.1%) were overweight or obese. Approximately 38% of the patients had a history of abdominal surgery. The majority of patients had an ASA score of II (66.1%) or III (27.5%).

**Table 1:** Patient characteristics and surgical indication (n=109) BMI - body mass index; ASA - American Society of Anesthesiologists

Variable	Value	%
Age (years)	55 (23–85) [45–63]	
BMI(kg/m²)	26.6 (15.6–49) [24–33]	
Overweight or obese (Yes/No)	71/38	65.1/34.9
Hypertension (Yes/No)	34/75	31.2/68.8
Diabetes(Yes/No)	10/99	9.2/90.8
Previous abdominal surgery (Yes/No)	41/68	37.6/62.4
ASA score		
I	7	6.4
II	72	66.1
III	30	27.5
IV	0	0
Indication		
Adnexal tumor	46	42.2
Endometrial tumor or precursors	33	30.3
Abnormal uterine bleeding	18	16.5
Cervical tumor or precursors	6	5.5
Uterine tumor	2	1.8
Surgical castration	2	1.8
Pelvic organ prolapse	2	1.8
Surgery		
Total hysterectomy	76	69.7
- with bilateral adnexectomy	56	
- with bilateral salpingectomy	13	
- with unilateral adnexectomy	6	
- with unilateral salpingectomy	3	
- with unilateral/bilateral pelvic lymphadenectomy ± para-aortic lymphadenectomy	7	
- with sentinel lymph node biopsy	13	
- with omentectomy	7	
- with peritoneal biopsies	5	
Unilateral or bilateral adnexectomy	20	18.3
- with omentectomy	1	
Unilateral cystectomy	8	7,3
- with unilateral or bilateral salpingectomy	2	
Bilateral oophorectomy	2	1,8
Bilateral salpingectomy	1	0,9
Subtotal hysterectomy + bilateral adnexectomy + sacrocervicopexy	1	0,9
Sacrocolpopexy	1	0,9

The main surgical indication was adnexal tumor in 46 patients (42.2%), followed by endometrial tumor or precursors in 33 (30.3%) and abnormal uterine bleeding in 18 (16.5%). The most common procedure was hysterectomy in 76 patients (69.7%), most of whom included concomitant adnexectomy. This was followed by adnexectomy (unilateral or bilateral) in 20 patients (18.3%) and cystectomy in eight (7.3%). In the context of pelvic organ prolapse, one sacrocervicopexy and one sacrocolpopexy were performed.

Intraoperative and postoperative outcomes are presented in Table 2. The overall median operative time was 194 minutes (range: 55-420). For procedures that included a hysterectomy, the median operative time was 200 minutes (range: 105-420). Regarding blood loss, most patients (66.1%) lost less than 100 mL of blood. Conversion to laparotomy occurred in a single patient (0.9%) due to technical difficulties caused by a significantly enlarged myomatous uterus. The median length of hospital stay was one day (range: one to six days). Two intraoperative complications occurred (1.8%): a bladder laceration, identified and corrected during the same surgery, and a right ureter injury, diagnosed on the first postoperative day and managed with percutaneous nephrostomy. Postoperative complications occurred in 11 patients (10.1%), 81.8% of which were of low severity (Grade I/II), and two were Grade III (1.8%): bilateral pulmonary thromboembolism, diagnosed on the seventh postoperative day and treated with in situ fibrinolysis, and the previously described ureteral injury. Three patients (2.8%) required readmission, without the need for reintervention: one vaginal vault hematoma treated conservatively, one case of vaginal vault bleeding (managed with the application of a hemostatic sponge and vaginal packing for 24 hours), and the previously described case of pulmonary thromboembolism. No mortality cases were recorded.

**Table 2:** Intraoperative and postoperative outcomes (n=109)

Variable	Value	%
Surgical time (median, minutes)	193.64 (55–420) (139–240)	
Estimated blood loss (mL)		
<50	43	39.5
50-99	29	26.6
100-199	19	17.4
200-499	6	5.5
≥500	3	2.8
No information available	9	8.3
Need for conversion to laparotomy	1	0.9
– Enlarged uterus with limited visualization of the right uterine artery		
Intraoperative complications	2	1,8
– Iatrogenic bladder laceration	1	0.9
– Iatrogenic right ureteral injury	1	0.9
Length of hospital stay (median, days)	1 (1-6)	
Need for readmission	3	2.8
– Vaginal vault hematoma	1	0.9
– Bleeding from vaginal vault	1	0.9
– Bilateral pulmonary embolism	1	0.9
Morbidity (Clavien-Dindo classification)	11	10.1
Grade I	6	5.5
Grade II	3	2.8
Grade III	2	1.9

**Figure 1:**
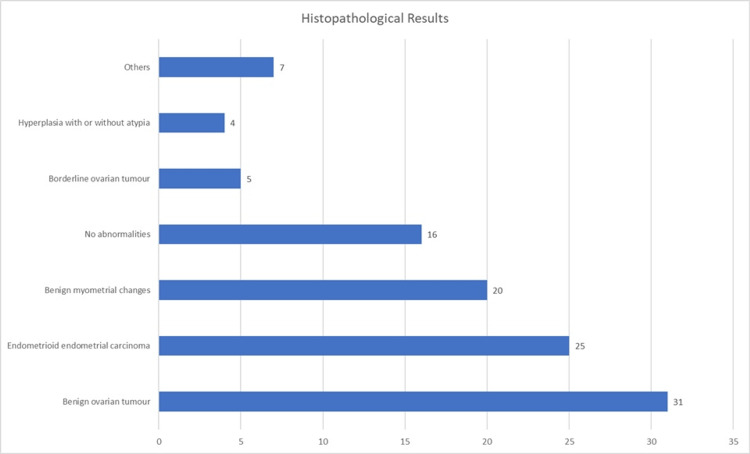
Histopathological results (n=108)

The histopathological results of the surgical specimens are described in Figure 1. The most frequent diagnosis was benign ovarian tumor, identified in 31 patients (28.7%), followed by endometrioid carcinoma of the endometrium in 25 patients (23.1%) and benign myometrial changes in 20 (18.5%).

## Discussion

Robotic gynecologic surgery has expanded globally and is now routinely used for various benign and malignant conditions, including hysterectomy, myomectomy, sacrocolpopexy, and endometriosis surgery [8].

Robotic surgery, with the aid of three-dimensional vision, provides a more detailed visualization of anatomical structures. The high precision and flexibility of the instruments allow for optimized access to anatomically challenging areas. Additionally, it offers ergonomic benefits for the surgeon and a shorter learning curve compared to conventional laparoscopy [9-12].

Limitations of this approach include the lack of tactile feedback, increased physical distance between the surgeon and the patient, and higher costs compared to conventional laparoscopy [9].

Robotic surgery has played a particularly important role in the treatment of obese patients, helping to overcome challenges such as trocar placement and the surgeon's limited mobility in larger abdomens [13]. Boggess et al. also identified reduced morbidity and shorter hospital stays in obese women undergoing robotic hysterectomy compared to other surgical approaches [14].

A Cochrane review that analyzed 12 randomized clinical trials on the efficacy and safety of robotic surgery versus conventional laparoscopy in benign and malignant gynecological conditions concluded that, for benign diseases, surgical complication rates are similar. However, the evidence related to malignant diseases in this review is less robust due to limited survival data [9,15]. Nevertheless, a recent randomized clinical trial evaluating overall survival, progression-free survival, and long-term surgical complications in patients with endometrial cancer who underwent either robotic surgery or conventional laparoscopy showed a slight benefit in overall survival in the robotic group, with no differences in progression-free survival [16].

Compared to laparotomy, robot-assisted procedures allow for faster recovery, shorter hospital stays, and significantly reduced blood loss, transfusion requirements, and postoperative pain [13,17,18]. A prospective study comparing a cohort of 1000 women undergoing robotic surgery and open surgery for endometrial cancer staging showed a higher rate of major complications, such as intraoperative hemorrhage, severe vascular injury, infection, paralytic ileus, and deep vein thrombosis, in the open surgery group. Furthermore, admissions to intensive care units and mortality rates were significantly lower in the robotic surgery group. The study also highlighted that, among obese patients, the incidence of complications was substantially lower with robotic surgery compared to open surgery (3.7% vs. 31%, p<0.0001) [19].

The use of robotic surgery is discouraged in simple gynecologic procedures such as tubal ligation, ovarian cystectomy, and diagnostic laparoscopy, due to the absence of significant benefits. The American College of Obstetricians and Gynecologists (ACOG) and the Society of Gynecologic Surgeons (SGS) recommend its use only when integrated into more complex surgeries [10]. Nevertheless, some of these procedures were performed during the initial phase of our robotic program. This decision was primarily related to the surgical team's learning curve, as well as specific clinical factors such as obesity, prior abdominal surgeries, or complex adnexal pathology. In these situations, robotic surgery was considered a safe and appropriate option.

The present study aims to succinctly describe the process of implementing robotic surgery in gynecology, offering a descriptive overview of the cases as a means of knowledge sharing.

The most frequent individual surgical indication was adnexal tumors (42.2%), and total hysterectomy was the most commonly performed procedure (69.7%). This is explained by the cumulative number of cases related to uterine pathology, which included a variety of benign and malignant conditions requiring hysterectomy as part of the surgical management.

The median operative time was 193.6 minutes (range: 55-420), which aligns with values reported in the literature. This variation reflects the heterogeneity of procedures included in the study, ranging from simple adnexectomies to complex surgeries. Reported median durations for robotic hysterectomy range from 131 to 181 minutes [20-22]. In our cohort, surgeries that included hysterectomy had a median operative time of 200 minutes (range: 105-420). These frequently involved additional procedures, such as adnexectomy, omentectomy, and lymphadenectomy, limiting direct comparison. Data on the time allocated exclusively to the hysterectomy are not available.

Regarding intraoperative blood loss, most patients (66.1%) experienced losses of less than 100 mL, and none required blood transfusions, which aligns with the literature [23,24].

The conversion rate to laparotomy was 0.9%, supporting the appropriateness of the technique even in more complex scenarios. This figure falls within the range reported in several similar published studies [23-26].

In our department, the combination of robotic surgery with Enhanced Recovery After Surgery (ERAS) protocols resulted in a median hospital stay of one day (range: one to six days). This finding aligns with international studies that indicate shorter hospital stays and earlier discharge are associated with robotic surgery, especially when compared to open or even laparoscopic procedures [10,15]. The only patient with a six-day hospitalization had an iatrogenic injury to the distal right ureter and underwent a percutaneous nephrostomy on postoperative day two.

The intraoperative complication rate was 1.8%, involving two cases: one bladder injury corrected immediately, and the ureteral injury mentioned above. These are recognized events in minimally invasive pelvic surgeries. Compared with the literature, similar studies report intraoperative complication rates ranging from 0% to 8.9% [22-25,27]. A retrospective study including 3114 hysterectomies reported urologic injury rates of 0.92% for robotic, 0.90% for laparoscopic, 0.96% for abdominal, and 0.33% for vaginal hysterectomies. These findings suggest that robotic surgery carries a comparable risk of urologic injury to other minimally invasive approaches [28].

In the present study, the readmission rate was 2.8%, and postoperative outcomes showed an overall complication rate of 10.1% among patients undergoing robotic surgery. Of these complications, 81.8% were low grade (Clavien-Dindo grade I/II). Only 1.8% (two cases) were classified as grade III: the previously mentioned ureteral injury (grade IIIa) and a case of bilateral pulmonary embolism (grade IIIb). The pulmonary embolism case occurred on postoperative day seven in a 28-year-old patient with a BMI of 38 kg/m², who had undergone cystectomy and unilateral salpingectomy for a large adnexal mass, with an operative time of 135 minutes and no intraoperative complications. She was hospitalized for one day and used graduated compression stockings as recommended. In addition to recent surgery, she had other thromboembolic risk factors: active smoking, obesity, and use of combined oral contraceptives [29,30]. The event was treated with in situ fibrinolysis, without requiring intensive care.

Regarding histological diagnosis, among the 16 cases without pathological abnormalities on final histology, the surgical indications were as follows: eight cases of staging procedures following a prior histologic diagnosis of gynecologic tumors; three cases of microinvasive cervical cancer or high-grade intraepithelial lesion (HSIL) diagnosed on prior conization specimens without indication for repeat conization; two cases of persistent abnormal uterine bleeding in postmenopausal women, including tamoxifen-associated cases; two cases of bilateral salpingo-oophorectomy in women with metastatic breast cancer; and one case of suspected adnexal mass that was not confirmed histologically and was intraoperatively found to be retroperitoneal.

This study has limitations inherent to its retrospective and observational design, as well as the heterogeneity and small size of the sample. Additionally, it reflects the experience of a single center during the early phase of implementation, which may influence the results due to the learning curve. The lack of long-term follow-up data also limits the assessment of late complications or the impact on quality of life, although a patient satisfaction study is currently underway.

As a descriptive analysis, this study does not aim to compare different surgical approaches but rather to objectively evaluate the applicability of a new surgical technique and compare the outcomes with published data. The authors also intend that sharing both our implementation process and results may be helpful to other centers interested in launching robotic surgery programs in gynecology.

## Conclusions

The implementation of robotic surgery in gynecology at ULS São José has proven to be a safe and effective approach, demonstrating a low conversion rate to laparotomy, reduced morbidity, and significantly shorter hospital stays. These positive outcomes align with international findings that highlight the advantages of robotic surgery, particularly in complex cases, such as those involving patients with obesity. Enhanced precision, improved visualization, and better surgical control contribute to more accurate, minimally invasive procedures, ultimately leading to faster recovery and fewer complications.

A key factor in the program's success was the structured training provided to the surgical team, which included theoretical education, live surgery observation, and hands-on simulation. This comprehensive approach ensured the team was well prepared to perform robotic procedures with competence and confidence. Continued investment in training and regular evaluation of clinical outcomes will be essential to further improving and expanding robotic surgery within the institution. By documenting our early institutional experience, this study provides valuable insights that may support other centers in adopting robotic gynecologic surgery, particularly in settings where such programs are still emerging.
